# Mineral and Electrolyte Disorders With SGLT2i Therapy

**DOI:** 10.1002/jbm4.10242

**Published:** 2019-11-04

**Authors:** Giuseppe Cianciolo, Antonio De Pascalis, Irene Capelli, Lorenzo Gasperoni, Luca Di Lullo, Antonio Bellasi, Gaetano La Manna

**Affiliations:** ^1^ Department of Experimental, Diagnostic and Specialty Medicine (DIMES), Nephrology, Dialysis and Transplantation Unit, St. Orsola Hospital University of Bologna Bologna Italy; ^2^ Department of Nephrology and Dialysis V. Fazzi Hospital Lecce Italy; ^3^ Department of Nephrology and Dialysis Parodi‐Delfino Hospital Colleferro Italy; ^4^ Department of Research Innovation and Brand Reputation, ASST Papa Giovanni XXIII Bergamo Italy

**Keywords:** BONE HEALTH, DIABETES, ELECTROLYTES, MINERAL METABOLISM, SODIUM‐GLUCOSE COTRANSPORTERS

## Abstract

The newly developed sodium‐glucose cotransporter 2 inhibitors (SGLT2is) effectively modulate glucose metabolism in diabetes. Although clinical data suggest that SGLT2is (empagliflozin, dapagliflozin, ertugliflozin, canagliflozin, ipragliflozin) are safe and protect against renal and cardiovascular events, very little attention has been dedicated to the effects of these compounds on different electrolytes. As with other antidiabetic compounds, some effects on water and electrolytes balance have been documented. Although the natriuretic effect and osmotic diuresis are expected with SGLT2is, these compounds may also modulate urinary potassium, magnesium, phosphate, and calcium excretion. Notably, they have had no effect on plasma sodium levels and promoted only small increases in serum potassium and magnesium concentrations in clinical trials. Moreover, SGLT2is may induce an increase in serum phosphate, FGF‐23, and PTH; reduce 1,25‐dihydroxyvitamin D; and generate normal serum calcium. Some published and preliminary reports, as well as unconfirmed reports have suggested an association with bone fractures. Some homeostasis perturbations are transient, whereas others may persist, suggesting that the administration of SGLT2is may affect electrolyte balances in exposed subjects. Although current evidence supports their safety, additional efforts are needed to elucidate the long‐term impact of these compounds on chronic kidney disease, mineral metabolism, and bone health. Indeed, the limited follow‐up studies and the heterogeneity of the case‐mix of different randomized controlled trials preclude a definitive answer on the impact of these compounds on long‐term outcomes such as the risk of bone fracture. Here we review the current understanding of the mechanisms involved in electrolyte handling and the available data on the clinical implications of electrolytes and mineral metabolism perturbations induced by SGLT2i administration. © 2019 The Authors. *JBMR Plus* published by Wiley Periodicals, Inc. on behalf of American Society for Bone and Mineral Research.

## Introduction

Diabetic kidney disease (DKD) is the leading cause of end‐stage renal disease (ESRD) worldwide and contributes to the increased cardiovascular morbidity and mortality, lower quality of life, and increased health care costs of patients with type 2 diabetes (T2D).^1^

Prevention of diabetic nephropathy (DN) by either preventing disease onset or progression of established chronic kidney disease (CKD) is a crucial therapeutic goal. Although glucose control remains the mainstay of treatment in patients with diabetes, renin–angiotensin–aldosterone system (RAAS) blockade is, to date, the only proven effective treatment for albuminuria reduction, as well as disease progression attenuation in DN.^2^

Great efforts have been devoted in recent years to developing multiple novel agents to tackle DN. However, irrespective of promising preclinical data, new drugs have frequently been proven less effective or tolerated than standard therapies in humans.^2^, ^3^ Of the recently approved oral hypoglycemic agents, the sodium‐glucose cotransporter 2 inhibitor (SGLT2i) compounds have been shown to improve both renal and cardiovascular outcomes.

As seen with other antidiabetic compounds, glucose metabolism manipulation by SGLT2is is associated with changes in electrolytes and water balance. We herein summarize clinical data on the use of SGLT2is and their effect on renal physiology, as well as their potential clinical effects in CKD patients.

## SGLT2i Use: Clinical Scenario

**Figure 1:**
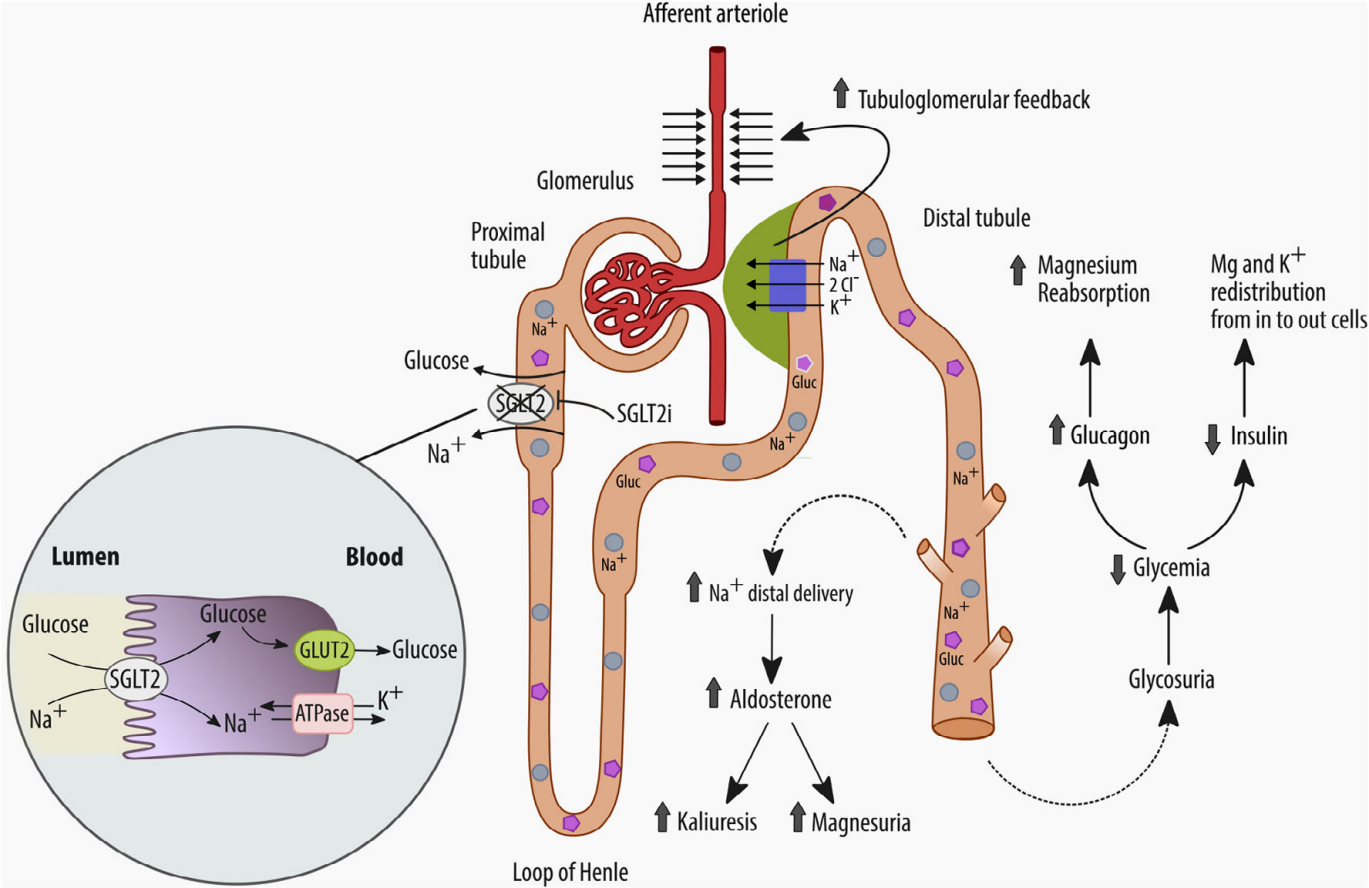
Postulated effects of SGLT2is on serum electrolytes (sodium, potassium, and magnesium): inhibition of SGLT2 receptors promotes glycosuria, natriuresis, and osmotic diuresis, which in turn causes an elevation of aldosterone activity with increased kaliuresis and magnesuria. These effects are counterbalanced by an improvement in glycemic control with an elevation of serum glucagon and reduction of insulin, which favors redistribution of potassium and magnesium in cells from the intracellular space. The net effect is a potential low increase of serum potassium and magnesium concentrations.

Currently, canagliflozin, dapagliflozin, empagliflozin, and ertugliflozin are the drugs of this class approved for use in the United States and Europe. SGLT2is block the reabsorption of glucose and sodium in the S1 segment of the proximal tubule, thereby augmenting urinary glucose and sodium excretion (Fig. 1). Inhibition of urinary glucose reabsorption results in a reduction in plasma glucose and hemoglobin A1c (HbA1c). However, reduction of sodium reabsorption, on the other hand, leads to increased delivery of sodium to the macula densa, which in turn stimulates the tubuloglomerular feedback and vasoconstriction of the glomerular afferent arteriola. Consequently, it reduces glomerular hyperfiltration, a mainstay of DN treatment. Of note, the decline in the glomerular filtration rate (GFR) is completely reversible after drug discontinuation.^4^

A favorable impact of these compounds on hard clinical outcome, such as cardiovascular and renal events, has been documented by two recently published large randomized clinical trials^4^, ^5^ and is under investigation in several other trials currently in progress. In the (empagliflozin) Cardiovascular Outcome Event Trial (EMPAREG OUTCOME trial), SGLT2 inhibition with empagliflozin reduced the primary outcome of major adverse cardiovascular events (MACEs), mortality, hospitalization for heart failure, as well as the progression of DKD.^4^, ^5^ In the Canagliflozin Cardiovascular Assessment Study (CANVAS) program trials, the use of canagliflozin reduced the rate of the major cardiovascular endpoint (MACE) and the risk of heart and renal failure. Nevertheless, an unexpected increased risk of lower extremity amputations and fractures was also reported.^6^ In the Dapagliflozin Effect on Cardiovascular Events–Thrombolysis in Myocardial Infarction 58 Study (DECLARE–TIMI 58), though no effect on MACE was noted, the administration of dapagliflozin resulted in a slower rate of cardiovascular death or hospitalization for heart failure.^7^ A positive impact of dapagliflozin on renal function was also documented in a post hoc analysis of 166 patients with stage 3 CKD and increased albuminuria.^5^ This study examined the long‐term effects of 2‐year treatment with dapagliflozin on urinary albumin/creatinine ratio (UACR) and showed a dose‐dependent reduction of 53.6% and 43.8% in UACR with 10 mg and 5 mg doses of dapagliflozin, respectively.^5^

Although promising, none of these studies considered kidney events as primary endpoints nor were the studies designed to provide definitive information on the potential reno‐protection associated with SGLT2is. Instead, all these studies were primarily designed to verify cardiovascular safety of SGLT2is and were performed in a selected population of patients with prior cardiovascular events and/or with very high cardiovascular risk. Before these agents can be routinely used as first‐line agents in patients with DKD, ad hoc and adequately designed clinical trials are needed. In this regard, the recently published Canagliflozin and Renal Endpoints in Diabetes with Established Nephropathy Clinical Evaluation Trial (CREDENCE) expands our understanding by showing a significant reduction of 30% (HR, 0.70; 95% CI, 0.59 to 0.82) in the risk of the composite endpoint of ESRD (defined as dialysis, transplantation, or a sustained estimated GFR [eGFR] of <15 mL/min/1.73 m^2^) in patients with T2D and albuminuric CKD (eGFR between 30 to 90 mL/min/1.73 m^2^) among patients treated with canagliflozin versus placebo.^8^Of note, no difference in rates of amputations or fractures was noted between study arms. Nevertheless, the trial was stopped early (median follow‐up 2.6 years) because of the favorable risk‐to‐benefit ratio of canagliflozin on renal function, limiting the statistical power of the analyses of these secondary outcomes.^8^ Furthermore, the question of whether these effects can be expanded to apply to more advanced stages of CKD remains unanswered.

Although the glycosuric effect is attenuated in patients with reduced renal function, SGLT2i‐lowering effects on blood pressure and albuminuria, as well as SGLT2i impact on eGFR, seem preserved in patients with moderate CKD. Data suggest that SGLT2is may have reno‐protective effects even in people with reduced kidney function in whom the glycemic effect is limited, and hence in whom other mechanisms beyond glucose‐lowering are likely involved.^9^ Understanding what mechanisms convey reno‐protection is of great importance because the use of SGLT2is may also potentially exert a positive effect on kidney function in nondiabetic patients with CKD. This hypothesis is currently being investigated in trials, such as A Study to Evaluate the Effect of Dapagliflozin on Renal Outcomes and Cardiovascular Mortality in Patients With Chronic Kidney Disease Study (DAPA‐CKD),^10^ which will provide important additional information regarding the safety of SGLT2is in nondiabetic CKD patients.

Although SGLT2is are generally well‐tolerated, they have been associated with some side‐effects that should be considered to optimize their risk‐to‐benefit profile in daily clinical practice. The most important adverse effects of SGLT2is (class effect) are genital and urinary tract infections and diabetic ketoacidosis, as well as volume‐depletion‐related issues caused by glycosuria and osmotic diuresis. Other adverse effects are an increase in fracture risk and electrolyte imbalances, in particular, mineral metabolism abnormalities.

Although the US Food and Drug Administration and the European Medicines Agency have both raised concerns about a potential increase in the risk of acute kidney injury (AKI) with SGLT2i use, the eGFR drop reported after SGLT2i administration may be caused by the acute and reversible reduction of GFR, reduction in the blood volume induced by the osmotic diuresis, and restoration of the tubulo‐glomerular balance.^11^ In the CANVAS trial, no increased risk of AKI (3.0 versus 4.1 events per 1000 patient‐years) or renal‐related adverse events (19.7 versus 17.4 events per 1000 patient‐years) was noticed even though volume depletion was more common in the canagliflozin group (26 versus 18.5 events per 1000 patient‐years; *p* = 0.009).^12^Similarly, in the EMPA‐REG OUTCOME study,^4^ the incidence of acute renal failure was lower in participants receiving empagliflozin when compared with those receiving placebo (5.2% versus 6.6% in the empagliflozin and placebo groups, respectively). Additionally, in a propensity‐matched analysis, Nadkarni and colleagues found that the risk of AKI among 377 SGLT2i users was about twofold lower when compared with 377 matched SGLT2i nonusers (3.8% versus 9.7% in users and nonusers, respectively; HR, 0.4; 95% CI, 0.2 to 0.7; *p* = 0.01).^13^ In a second and larger cohort of 1207 SGLT2i users and 1207 propensity‐matched SGLT2i nonusers, the same authors observed that the frequency of AKI was 2.2% and 4.6% (HR, 0.5; 95% CI, 0.3 to 0.8; *p* < 0.01) in users and nonusers, respectively.^14^ Although volume depletion following SGLT2i use does not seem to cause AKI overall, it may precipitate renal failure in some specific clinical settings, such as with elderly people treated with RAAS inhibitors and/or high doses of diuretics.

These topics may be relevant especially in the context of CKD and long‐term exposure to these drugs. Although, available evidence suggests that chronic administration of these compounds is associated with some degree of nephro‐protection (a decrease in albuminuria and decrease in the rate of the decline of renal function) caused by a reduction of glomerular hyperfiltration and intraglomerular pressure, the EMPAREG, the CANVAS, and the CREDENCE studies enrolled a limited number of patients with advanced CKD (all studies excluded individuals with eGFR <30 mL/min/1.73 m^2^) and high cardiovascular risk profile. Although it is uncertain if these results are generalizable to other patient subgroups and whether SGLT2i hypoglycemic effects are preserved in advanced CKD, extra effort is needed to elucidate the real impact of SGLT2is in these patients.^4^, ^6^

Summaries of the product characteristics of these drugs suggest that empagliflozin and canagliflozin should be discontinued when eGFR falls below 45 mL/min/1.73 m^2^ and 60 mL/min/1.73 m^2^, respectively. Of note, none of the published trials investigated the clinical relevance of electrolyte and mineral metabolism imbalances.

The aim of this article is to review available evidence on the effects of SGLT2is on renal tubules, electrolyte imbalances, and the potential clinical effects of these drugs on CKD patients.

## Clinical and Preclinical Studies on Electrolytes Imbalances

In consideration of the renal site of action and the associated osmotic diuresis, there is general concern about the potential effects of SGLT2is on electrolyte balances (including sodium, potassium, magnesium, calcium, and phosphate) and volume depletion. However, despite the fact that they might trigger volume depletion and stimulate the tubule‐glomerular feedback, available evidence suggests that SGLT2is are associated with mild changes in serum electrolyte levels. Indeed, no effect on plasma sodium levels and only small increases in serum potassium, calcium, phosphate, and magnesium concentrations have been found in clinical trials.^15^, ^16^, ^17^

### Effects on sodium

Though a negative sodium balance may be postulated during SGLT2i administration, no changes in serum sodium levels have been reported in clinical studies despite signs of hemoconcentration.^18^ Although mild in degree, sodium and water depletion may contribute to some positive actions of SGLT2is (ie, reduction of blood pressure, possible reduction of hospitalizations for heart failure), but evidence is far from being conclusive.

In healthy human kidneys, the proximal tubule reabsorbs 60% to 80% of the sodium filtered by the glomeruli every day. The SGLT2 receptor plays a significant role in this process, reabsorbing from the lumen of the early proximal tubule one sodium ion (Na^+^) for every glucose molecule (Fig. 1). The remaining filtered sodium load is reabsorbed in the late proximal tubule by the high‐affinity (two Na^+^ ions for every glucose molecule) SGLT1 receptors in the S2/S3 segments of the proximal tubule. However, in healthy subjects the latter is underused because over 90% of the filtered glucose is reabsorbed via SGLT2.

In diabetic individuals, the increased expression and activity of SGLT2 and full recruitment of SGLT1 cause increased sodium reabsorption in the proximal tubule. Enhanced sodium reabsorption leads to reduced sodium uptake at the macula densa level, activation of the tubulo‐glomerular feedback pathway, and reduction of the synthesis of vasoconstrictive molecules acting on afferent arteriola that leads to increased renal plasma flow and intraglomerular capillary hydrostatic pressure. All these factors are responsible for glomerular hyperfiltration, a precursor of intraglomerular hypertension and DKD.

SGLT2 inhibition exerts opposite effects. Sodium and glucose, which are not absorbed proximally via SGLT2, pass distally because other sodium transporters, such as SGLT1 or Na+/H+ exchanger‐3‐ (NHE3‐) dependent ones, localized along the nephron, only partially compensate for the reduced absorption of sodium operated by SGLT2. Hence, SGLT2 inhibition results in the increased delivery of sodium to the macula densa, which in turn stimulates the vasoconstriction of the glomerular afferent arteriola and reduced glomerular hyperfiltration, as well as GFR in the first weeks of treatment.

Though evidence suggests that SGLTis are nephro‐protective, to the best of our knowledge, no study has investigated how these compounds change sodium balance. Studies are needed to elucidate the clinical relevance of natriuresis and osmotic diuresis in selected patients at high risk of dehydration, such as the elderly and CKD patients.

### Effect on potassium

Diabetes portends a high risk of hyperkalemia and is often associated with a variable degree of renal function impairment.^14^ Furthermore, the frequent use of drugs affecting the RAAS also contributes to the development of hyperkalemia in diabetic patients.^19^, ^20^ In this regard, some but not all studies have also reported a slight increase in serum levels of potassium with SGLT2is. In particular, a small increase in serum potassium has been reported with the use of canagliflozin, but not with empagliflozin and dapagliflozin. In a pooled analysis of four placebo‐controlled studies using canagliflozin, an association with dose (100 versus 300 mg/day) and renal function (eGFR ≥60 mL/min/1.73 m^2^ versus eGFR ≥45 and < 60 mL/min/1.73 m^2^) was reported. The occurrence of serum potassium levels >5.4 mEq/L at any time during follow‐up was 4.5%, 6.8%, and 4.7% in patients treated with 100 mg and 300 mg of canagliflozin and placebo, respectively. Patients with more‐advanced CKD presented an almost 1.3‐fold higher incidence of hyperkalemia (9.1% versus 6.8%) when treated with 300 mg of canagliflozin. Of note, other factors, such as the use of antihypertensive agents that affect potassium excretion or acidosis, were also associated with hyperkalemia in patients receiving or not receiving canagliflozin.^21^ In the EMPA‐REG OUTCOME trial, the use of empagliflozin in patients with T2D and mild CKD (stage 2 and 3) was not associated with changes in serum potassium levels.^22^ Similarly, dapagliflozin was not associated with serum potassium changes in patients with moderate renal impairment (eGFR 30 to 59 mL/min/1.73 m^2^).^23^ These reassuring findings were also confirmed by a pooled analysis of 14 randomized clinical trials, which showed that dapagliflozin was not related to an increased risk of hyperkalemia, even in patients with baseline eGFR between 30 and 60 mL/min/1.73 m^2^.^16^ Of interest, at least in this pooled analysis, the risk of hyperkalemia was similar among patients treated with angiotensin‐converting enzyme inhibitors, angiotensin receptor blockers (ARBs), or potassium‐sparing diuretics, even those with moderate renal impairment, supporting the notion that dapagliflozin is not associated with serum potassium‐level abnormalities.

It is not clear whether this effect on potassium is, in part, dependent on small changes in GFR or volume status. It is possible that a slight reduction in volume status leads to a reduction in sodium delivery to the cortical collecting duct in the distal nephron, resulting in less sodium exchanged for potassium. Also, the decreased insulin concentrations observed with SGLT2i administration (caused by decreased glucose levels and the subsequent improvement in insulin resistance) may induce potassium redistribution from the cells to the extracellular space and contribute to the increased serum potassium levels reported in some studies.^24^

Independently of the mechanism, the different incidences of hyperkalemia in the SGLT2 trials possibly reflect a difference in case‐mix of the study cohorts (associated comorbidities such as renal impairment) and the use of drugs that potentially affect potassium homeostasis, such as inhibitors of the renin–angiotensin system. Therefore, even though the increase in serum potassium concentration is small, special care and potassium monitoring seems indicated in patients with renal impairment, in patients receiving concomitant anti‐RAAS medications, or in those affected by medical conditions predisposing to hyperkalemia.^25^

### Effects on magnesium

Tang and Zhang in a meta‐analysis of 18 randomized controlled trials, which included 15,309 patients with four SGLT2is (canagliflozin, empagliflozin, dapagliflozin, and ipragliflozin), showed that these drugs can increase serum magnesium levels by approximately 0.08 to 0.2 mEq/L in diabetic patients without normal renal function. Interestingly, canagliflozin increases serum magnesium in a linear and dose‐dependent manner.^17^ These findings are also confirmed by another analysis showing a dose‐dependent increase in serum magnesium levels along with increases in serum potassium and phosphate levels with canagliflozin administration. Of importance, these effects were larger in individuals with reduced renal function.^21^ Additionally, a post hoc analysis based on pooled data from four randomized controlled trials (RCTs) of canagliflozin showed that more patients with baseline serum magnesium below <0.74 mmol/L presented a normal serum magnesium level at study completion when treated with canagliflozin.^26^

Many mechanisms may explain the increase in serum magnesium levels induced by SGLT2i use.^27^ It is well‐known that hypomagnesemia caused by renal magnesium wasting is often associated with diabetes mellitus.^28^ Magnesiuria is likely caused by the reduced activity of the transient receptor potential melastatin 6 (TRPM6) ion channel in the distal convoluted tubules and is possibly related to insulin resistance.^29^, ^30^ In fact, a downregulation of TRPM6 channels, hypermagnesiuria, and hypomagnesemia were observed in obese T2D rats.^31^ Hence, the improvement of insulin resistance observed after SGLT2i administration may be associated with reduced magnesium excretion through TRPM6. The extracellular volume depletion induced by natriuresis and osmotic diuresis can also lead to a small increase in serum magnesium caused by hemo‐concentration.^27^ Also, the increased glucagon concentrations commonly observed during SGLT2i administration can also affect magnesium homeostasis because glucagon is associated with increased magnesium reabsorption in the distal renal tubules.^32^, ^33^ Finally, it should also be mentioned that insulin induces a shift of magnesium from the plasma to the intracellular space^34^ The reduced serum insulin levels observed after SGLT2i administration may contribute to a redistribution of magnesium out of the cells to the extracellular space. However, the effect of SGLT2is on magnesium is not completely understood because SGLT2is can also increase aldosterone concentrations caused by natriuresis and volume depletion. Aldosterone, in turn, may have a direct effect on magnesium transport, leading to increased magnesium excretion.^27^ In this regard, spironolactone, an aldosterone inhibitor, can be used to decrease renal magnesium wasting. Hence, it seems that the effect of SGLT2is on magnesium is the result of a complex balance of mechanisms that lead to an elevation or a decrease of serum magnesium concentrations, ultimately leading to a small increase in serum magnesium levels.

Although it could be postulated that the small increase in serum magnesium concentration may have contributed to the cardiovascular benefits shown in the EMPA‐REG OUTCOME and the CANVAS trials, the clinical relevance of SGLT2i effects on serum magnesium awaits confirmation.

## SGLT2i Effects on Bone Health and Mineral Metabolism

Preclinical and clinical studies designed to investigate the effects of SGLT2is on bone health suggest a complex scenario characterized by a derangement of mineral metabolism indexes and an impairment of bone strength.^35^, ^36^

The interpretation of these findings should take into account that diabetes involves derangements of the microarchitecture and the material properties of bone tissue: This condition is now referred to as diabetic bone disease (DBD). Similarly, the degree of renal impairment and its associated bone and mineral derangements (known as chronic kidney disease‐mineral and bone disorder [CKD‐MBD^37^) is another confounding factor that should be evaluated to understand the results of the ongoing trials aimed at identifying a possible long‐term effect on bone health and mineral metabolism of these drugs in diabetic patients with CKD.

### Diabetic bone disease

Increased bone fragility and risk of fracture are features of both type 1 diabetes and T2D. Indeed, the risk of fracture in diabetic individuals is greater than what is predicted by BMD.^36^, ^38^ The increased risk of fracture in these patients may be caused by poor bone quality, possibly because of greater cortical bone porosity and abnormal bone mineral strength.^39^ Poor bone quality and abnormal bone microarchitecture in DBD is likely the consequence of many common variables, including chronic hyperglycemia, tissue‐specific accumulation of advanced glycation end‐products (AGEs), abnormal insulin levels, and chronic low‐grade inflammation.^35^, ^36^ All these factors may be responsible for bone‐remodeling abnormalities in diabetes. The AGEs accumulation in collagen triggers apoptosis of the osteoblast cells, whereas abnormal serum levels of markers of bone formation, such as alkaline phosphatase, osteocalcin, insulin‐like growth factor‐1 (IGF‐1), sclerostin, wingless/integrated (Wnt) signaling pathways, and Runt‐related transcription factor 2 (Runx2) levels, suggest abnormal osteoblastogenesis.^36^ Similarly, abnormal serum levels of markers of bone resorption, such as receptor activator of NF‐κB ligand, tartrate‐resistant acid phosphatase, and c‐terminal cross‐linked telopeptide, suggest that impaired bone reabsorption may also be involved in the pathogenesis of skeletal fragility in diabetes.^39^ Chronic hyperglycemia and AGEs accumulation also contribute to inflammation, a pathophysiological hallmark of diabetes. Besides cardiovascular complications, any chronic inflammatory state can be expected to affect bone cell activity and bone health.^40^

Hence, all these metabolic derangements may predispose to mineral metabolism abnormalities and future efforts are needed to disentangle the impact of SGLT2is on bone health, particularly if advanced age and CKD coexist.

### Effects of SGLT2is on bone health and mineral metabolism indexes

Although data are still inconclusive, a growing body of evidence suggests that the use of SGLT2is may have some effects on bone health in specific subgroups of patients at high risk for fracture.

An increased incidence of bone fractures was first reported in a clinical study of dapagliflozin treatment in 252 patients with diabetes who had moderate renal impairment (eGFR <60 mL/min). During 104‐week follow‐up, 19 (7.7%) patients reported a bone fracture while receiving dapagliflozin, as opposed to no fractures among patients receiving a placebo. Of the 19 patients reporting fracture, 5 and 8 patients also presented with CKD stage 3A and 3B, respectively.^23^ However, this preliminary observation was not confirmed by a randomized double‐blind placebo controlled study, in which no significant differences in fracture risk were found between dapagliflozin and placebo over 50 weeks of treatment.^41^ Of note, in this study only 6 of 180 (3.3%) enrolled patients had an eGFR between 59 and 30; no patients had an eGFR <30 mL/min.

The CANVAS program (including CANVAS and CANVAS‐R studies) confirmed this safety signal. Indeed, the rate of all bone fractures was higher in patients treated with canagliflozin when compared with patients treated with placebo (15.4 versus 11.9 per 1000 person‐years; HR, 1.26; 95% CI, 1.04 to 1.52), and no difference was noted when low‐ and high‐energy trauma fractures were analyzed separately. These results were observed in the CANVAS trial, but not in the CANVAS‐R trial, suggesting that differences in renal function and in the case‐mix participants enrolled in the latter trial might be considered at higher fracture risk owing to their greater degree of renal function impairment.^6^, ^42^ A similar conclusion was also reached after a pooled analysis of 10 different RCTs: An increased risk of fracture (mainly driven by the CANVAS results) was associated with canagliflozin treatment in older patients at high risk for cardiovascular disease with lower eGFR and higher diuretic use at the study's inception.^43^ If this safety signal was detected in subgroups of patients, a large observational study of more than 150,000 diabetic patients found that treatment with canagliflozin had similar overall odds of bone fracture (defined as the composite endpoint of humerus, forearm, pelvis, or hip fracture requiring intervention) when compared with glucagon‐like peptide‐1 agonist.^44^

Data on empagliflozin suggest that this SGLT2i may have minimal or no effect on bone metabolism. Indeed, in the EMPAREG OUTCOME study the proportion of participants who developed fractures was equally low in both the empagliflozin and the placebo group (3.8% and 3.9%, respectively).^4^, ^22^ This result is supported by the results of a pooled analysis^45^ of 8500 diabetic patients from 17 placebo‐controlled (phase I to III trials) studies and 6 extension trials (study duration up to 2 years), which found no significant changes in bone biomarkers among empagliflozin‐treated patients. The limited overall impact of these compounds on bone health is also supported by four different meta‐analyses of RCTs evaluating the safety outcomes of canagliflozin, dapagliflozin, and empagliflozin treatment; there was no harmful effect of SGLT2is on bone health.^43^, ^46^, ^47^, ^48^

Future studies should evaluate whether the positive effects of these drugs on renal function overcome or counterbalance in the long term the potential adverse effects on bone health.

Studies assessing markers of bone turnover and BMD as surrogates for bone health in patients treated with SGLT2is have yielded contradictory results. In a RCT, Bilezikian and colleagues showed that the administration of 100 or 300 mg of canagliflozin for about 2 years was associated with a decrease in total hip BMD. The authors also reported a significant increase in the serum levels of markers of bone resorption (such as collagen type 1 β‐carboxy‐telopeptide) and formation (such as osteocalcin), suggesting a possible effect of canagliflozin on bone metabolism.^49^

In contrast to canagliflozin, no effect of dapagliflozin on BMD or serum biomarkers of bone turnover in patients with normal to mildly impaired renal function has been reported. This raises the question of whether these differences are because of the case‐mix of the studies or whether the effects on bone metabolism are compound‐specific.^50^

This body of evidence, though reassuring, is likely still too limited to address the safety issue in high‐risk subgroups of patients such as those with advanced CKD. Indeed, the limited study follow‐up (that ranged from 6 months to 2 years) and the heterogeneity of the case‐mix and study design of different RCTs preclude a definitive answer on the impact of these compounds on a long‐term outcome, such as the risk of fracture. Similarly, studies that utilize surrogate markers of bone health cannot completely rule out safety concerns because of their weak correlation with the risk of bone fracture.

In addition, uncertainty arises from animal model studies that also support the notion that SGLT2is can potentially affect bone health. In two separate studies,^35^, ^36^ it was reported that treatment with canagliflozin of DBA/2 J mice, a model of DBD, was associated with increased bone resorption and derangements of cortical and trabecular bone microarchitecture as well as bone strength. Additionally, trabecular bone abnormalities were also noted in canagliflozin‐treated control (nondiabetic) mice. The effects on bone tissue were coupled with changes in bone biomarkers. Mice treated with canaglifozin exhibited increased calciuria (because of the osmotic diuresis triggered by glycosuria) together with a rise in the serum levels of FGF‐23 and PTH. Changes in gene expression (about an 11‐fold increase) of renal CYP27B1 and sodium‐dependent phosphate transporter 2A (about a 30% decrease) were also noted.^35^, ^36^ Whereas the former is a key factor in vitamin D metabolism, the latter regulates renal phosphate handling.^36^

Recently, two studies have tried to shed light on the mechanisms involved in the changes of bone biomarkers associated with SGLT2i therapy. In a study conducted of healthy volunteers, treatment with canagliflozin acutely (within 24 to 72 hours of therapy initiation) increased serum phosphate by 16%, FGF‐23 by 20%, and PTH by 25%, as well as decreased 1,25‐dihydroxyvitamin D by 10%, compared with controls. According to these findings, the increase in serum phosphate in response to canagliflozin administration was caused by a significant increase in renal tubular reabsorption of phosphate. Hence, the increase in plasma FGF‐23 was a physiological response to the increase in serum phosphorus, which possibly explains the decrease in plasma 1,25‐dihydroxyvitamin D.^51^

The result of a secondary analysis of a crossover double‐blinded, placebo‐controlled trial further corroborates this hypothesis. Indeed, De Jong and colleagues^52^ reported on small albeit significant increases in serum phosphate (9%), FGF‐23 (19%), and PTH (16%) in a cohort of 31 patients with T2D complicated by CKD stages 2 to 4 and albuminuria who were treated with dapagliflozin for 6 weeks. Notably, these changes were mirrored by a significant 12% decrease in 1,25‐dihydroxyvitamin D; no effect on tubular reabsorption of phosphate and serum calcium was noted.^52^

**Figure 2:**
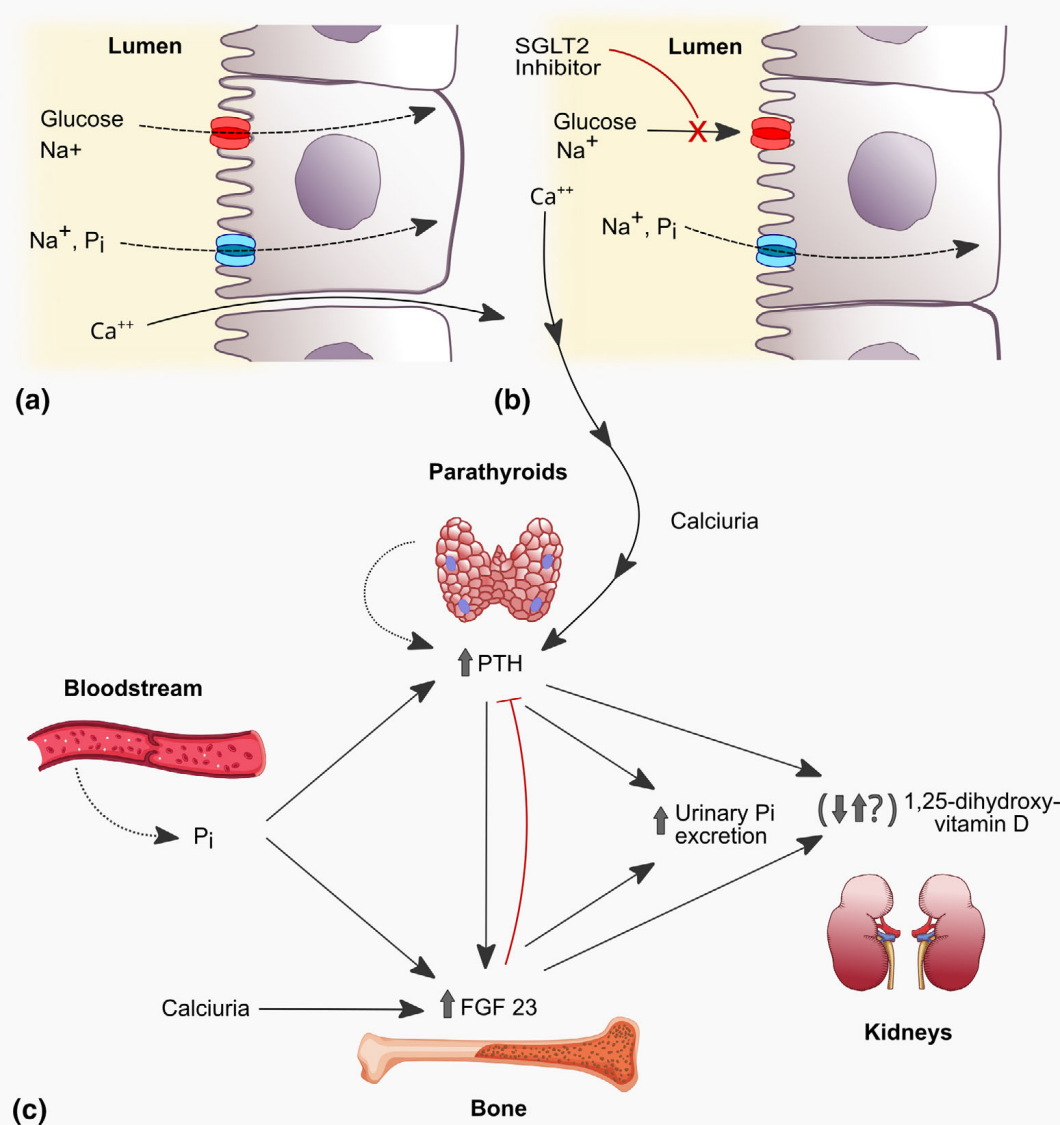
**Putative pathways of mineral imbalances and related‐hormones following SGLT2i therapy.** A) Sodium, glucose, phosphate and calcium reabsorption in proximal tubule under physiological conditions. B) By inhibiting cotransport of both Na^+^ and glucose, SGLT2i increases Na^+^ availability in the proximal tubule where sodium‐dependent phosphate cotransporters NaPi‐2a and NaPi‐2c are expressed. This, in turn, increases the electrochemical drive to reabsorb phosphate. C) Enhanced phosphate reabsorption and elevated serum phosphate levels trigger increases FGF23 secretion that accounts for suppressed 1,25‐dihydroxyvitamin D. Low 1,25‐dihydroxyvitamin D levels contribute to PTH increase both directly, via reduced feedback inhibition, and indirectly by reducing dietary calcium absorption and serum levels of calcium. SLGT2i also increases urinary Ca^2+^ excretion, that, in turn, may trigger the increase of both PTH and FGF23.

To reconcile available evidence, it is plausible that the primary trigger of the changes of mineral metabolism following SGLT2is entails the increased phosphate‐dependent sodium reabsorption in the proximal tubule caused by decreased glucose‐dependent sodium reabsorption. By inhibiting the cotransport of both Na^+^ and glucose, SGLT2is increase Na^+^ availability in the regions of the proximal tubule where NaPi‐2a and NaPi‐2c are expressed and modulate renal phosphate handling. The elevated intraluminal Na^+^ concentration, in turn, increases the electrochemical drive to reabsorb phosphate (Figs 2A and 2B). Higher serum phosphate levels induce the osteocyte synthesis of FGF‐23 that inhibits renal expression of CYP27B1 and 1,25‐dihydroxyvitamin D conversion. Low calcitriol levels are, in turn, responsible for high PTH levels directly via reduced feedback inhibition, and indirectly by reducing dietary calcium absorption (Fig. 2C).^51^, ^52^ The combination of decreased 1,25(OH)2D and increased PTH and FGF‐23 levels likely contribute to the increased fracture risk associated with SGLT2is.

Although this hypothesis may explain how SGLT2is may be associated with an increased risk of fracture, further efforts are needed to better elucidate the mechanisms underlying these changes. In healthy volunteers treated with canagliflozin, Blau and colleagues^(52)^ found that 24‐hour urinary calcium excretion was decreased 2 days after the 1,25‐dihydroxyvitamin D levels nadir; this correlated with maximal increases in PTH. In healthy volunteers, an increase in serum phosphorus and FGF‐23 and a decrease in 1,25(OH)D2 are only transient, and these biomarkers trend back to baseline values. By contrast, phosphate and PTH elevation persist and is larger in patients with impaired renal function, suggesting that SGLT2is change mineral metabolism homeostasis.^53^

These findings were quite unexpected in light of the increased urinary calcium reported in rats and mice treated with SGLT2is.^54^, ^55^ Currently, it is unknown whether urinary calcium results from a direct effect on proximal Ca^2+^ reabsorption or from glucose‐induced osmotic diuresis.^54^, ^55^ However, increased urinary calcium may be regarded as a further potential trigger for both PTH and FGF23 secretion because animal data suggest that these two phosphaturic hormones also act as calcium‐conserving hormones in the distal nephron (Fig. 2C).^56^

These complex feedback pathways may account for the large standard deviation of 1,25‐dihydroxyvitamin D and PTH reported in different studies. This may preclude the prediction of the net effect of SGLT2is on mineral metabolism as well as the long term clinical consequences also considering that changes in phosphate homeostasis may persist over the treatment period.^51^, ^52^, ^53^, ^57^

These effects may be of relevance for patients with DKD. These patients are susceptible to the complex scenario of CKD‐MBD, a disorder that following the development of hyperphosphatemia involves a progressive derangement of the FGF‐23–1,25(OH)2D–PTH axis, which is associated with an increased rate of bone fractures.^58^, ^59^, ^60^, ^61^

Whether the large variability among published studies is caused by different case‐mixes or other factors is currently unclear. Although it is possible that various SGLT2i compounds may exert potentially different effects on mineral metabolism and bone homeostasis, it is also possible that part of this intricate network of hormones may be progressively impaired as GFR declines. Whether SGLT2i‐induced bone mineral abnormalities are similar to those seen in normal renal function warrants confirmation.

## Conclusion

Modulation of renal SGLT2 is offers the opportunity to effectively control glucose metabolism in diabetes. Although clinical data suggest that these new compounds (empagliflozin, dapagliflozin, canagliflozin, ertugliflozin, ipragliflozin) are safe and protect against renal and cardiovascular events, little attention has been dedicated to the effects of these compounds on renal handling of different electrolytes. Although a natriuretic effect and osmotic diuresis are expected, these compounds may also modulate urinary potassium, magnesium, phosphate, and calcium excretion. Some of the homeostasis perturbations are transient, whereas others may persist, suggesting that the administration of these compounds may induce new electrolyte homeostasis in treated patients. Similarly, the influence of SGLT2is on bone health has been poorly investigated. What are the subtle mechanisms involved? Do all the compounds exert similar effects? May these derangements lead to adverse effects with prolonged use, especially in high‐risk patients such as those with renal dysfunction? These key questions remain matters of discussion requiring further studies.

## Disclosures

Authors declare no conflict of interest and no funding.
